# Exergy, economic, and environmental study for flat plate solar collector using pulsating flow

**DOI:** 10.1038/s41598-025-30498-0

**Published:** 2025-12-23

**Authors:** M. A. Sharafeldin, S. A. Marzouk, Mohamed T. Abdelghany

**Affiliations:** 1 Department of Mechanical Engineering, Faculty of Engineering at Shoubra, Benha University, Benha, Egypt; 2 Mechanical Engineering Dept, Faculty of Engineering, Kafr El-Sheikh University, Kafr El-Sheikh, Egypt; 3 High Institute of Engineering, October 6 City, Egypt

**Keywords:** Pulsating flow, Solar collector, Exergy efficiency, Economic, Environmental, Energy payback period, Energy science and technology, Engineering, Environmental sciences

## Abstract

Solar energy is considered a fundamental pillar in the principles of global sustainability for clean energy. Therefore, the main goal of researchers is to search for methods to improve the performance of solar collectors. Hence, this research aims to study the impact of pulsating flow on solar collectors through exergy, economic, and environmental studies. To make measurements, a comprehensive test system was developed. Pressure, flow rate, sun radiation, fluid temperature, and ambient temperature were all measured. Calculations were made for exergy efficiency and entropy generation. The cost of the heat that the system’s design generated was established. Savings on greenhouse gas emissions were calculated. The highest Exergy efficiency reached 9.33% for 4 Hz at a flow rate of 360 L/hr. The entropy generation was changed between 0.655 and 0.296 kW/k for continuous and pulsating flow, respectively. According to calculations, energy production may be increased by as much as 33.5%. The pulsing flow achieved a net reduction in CO2 emissions of 13.4 tons per year. In the present situation under study, the energy price to 0.063$/kWh. The energy payback period based on energy and exergy analysis was 4.34, 24.17 years, respectively.

## Introduction

Sustainable development is based on solar energy, which provides a clean and limitless substitute for traditional energy sources. By transforming solar radiation into usable thermal energy, solar collectors serve a critical role in this context, lowering greenhouse gas emissions and reducing reliance on fossil fuels. From the standpoint of engineering, solar collectors must be continuously improved in order to maximize heat transfer, increase energy efficiency, and guarantee system dependability in a variety of environmental circumstances. From an environmental perspective, their use encourages balance between environmental stewardship and technology growth and helps to preserve ecosystems. Hence, the following part provides the latest methods used to boost its usage. One method was changing the area shape, as Anthony et al.^1^ claimed. They discovered that the equal-sided square collector was more balanced and marginally compared with other shapes. According to Du et al.^2^, heat pipes with fins or grooves could significantly boost heat transfer. In the FPSC, Pathak et al.^3^ examined copper and aluminium as absorber plate materials. Spiral, wavy, and U-shaped pipes were among the several pipe shapes that were evaluated by Saffarian et al.^4^. They showed that wavy and spiral shapes had better thermal efficiency than simple U-shaped ones. Sundar et al.^5^ tested a collector with twisted tape inserts and nanofluid. The combined passive approach resulted in an 11.53% reduction in collector area compared to the standard collector. Czerwinski^6^ conducted experiments with and without pulsation. The term “pulsing” describes a flow that exhibits periodic changes. John R. Womersley was the first to derive it, hence the name Womersley Flow. More academics are now interested in analysing its effects on thermal energy applications. The impact of pulsation on the thermal energy and pressure drop of the CCT was examined by Abdelghany et al.^7^. Pulsating flow investigations were performed for all over a range of Dean numbers within 1148 and 2983, pulsing frequencies between 4 and 10 Hz, and Womersley numbers between 30 and 48, which translates to coil torsion between 0.02 and 0.052. Rouhollah Farhadi and Morteza Taki^8^ investigated the variables that affect the formation of shadows within a solar collector. Additionally, the loss in energy gain caused by shadows was calculated. Koukou et al.^9^ built and examined a new Integrated Collector Storage Solar Water Heater. The layout of the solar collector absorber plate was altered by Sharma et al.^10^. They evaluated the effectiveness of the absorber tubes’ trapezoidal and circular absorber surfaces. Ramesh and Sekar^11^ looked at the behaviour of a solar collector with a coated absorber plate that had glass reflectors at different angles. An experimental examination of the effects of different geometrical features of the solar collector on the effectiveness of the solar vortex engine was presented by Al-Kayiem et al.^12^. A tubular solar collector and two flat plate solar collectors were examined for energy absorption effectiveness by Ahmadlouydarab et al.^13^. Dhairiyasamy et al.^14^ looked at how adding horizontal obstructions to the internal cavity of FPSC affected their performance. Improving thermal performance primarily aims to reduce convective heat loss. An experimental examination bench was used to evaluate five solar collectors in a controlled setting. Fang et al.^15^ demonstrated how to connect a PV/T module at a fair price in order to increase the functional uniqueness of components. Atienza-Márquez et al.^16^ suggested an appropriate sizing approach for the solar thermal equipment utilized to deliver hot water. Sharafeldin and Abdelghany^17^ studied the effect of pulsating flow on the efficiency of a solar collector at different frequencies. Pulsating flow effects were predicted by Abdelghany et al.^18^ using artificial neural networks.

By using solar energy as an alternative source of energy in residential structures, Shaddel and Shokouhian^19^ aimed to reduce natural gas flaring. A solar thermal collector has been created to support this concept. Installed on the roof, this application is in charge of heating the water that circulates in radiator loops, providing space heating, or providing domestic hot water to meet washing demands in residential units. To calculate the expenses and establish the economic rationale for solar water heaters, Hoseini et al.^20^ examined residential apartment properties. The descriptive-analytic approach and scaling strategy used in their study fall under the category of progressive research undertaken in 2014.

Moghadam^21^ examined the technical and financial assessment of 300-litre solar water heater systems with natural movement using geographical and meteorological data from 31 Iranian provincial capitals. The payback period and the net present value of the solar system’s savings account were calculated. A new approach to figuring out the best orientation and size for solar water heating systems was described by Yılmaz^22^. Using the System Advisor Model, transient modelling was done to forecast system efficiency over a full year. One of the main drivers of the conventional energy transition to renewable energy has already been the fact that the inherent temporal intermittence and fluctuation of renewable energy sources, according to Shinnar^23^, like solar and wind, necessitates the development of effective and affordable energy storage systems. Spain, the United States, Egypt, Morocco, Mexico, Algeria, and Iran are all developing several projects. For solar thermal power facilities, the levelized cost of electricity (LCE) ranges from 13 to 21 €Cts/kWh, as per Eck and Hennecke^24^.

Sudhakar and Tara^25^ presented the findings of an initial techno-economic analysis of solar thermal power production at three sites in India. The System Advisor Model, created by NREL, USA, is used in the study. The study’s findings offer valuable information on (a) choosing the right reference normal direct sunlight for solar thermal power plant design, (b) figuring out the best mixes of solar multiple and thermal energy storage hours, and (c) potential cost savings. For manufacturing heating from solar thermal power plants, the levelized cost of thermal energy was found to be between 5 and 9 USD cents per kWhth, with significant sensitivity to collector efficiency and price as well as financial factors like taxation, discount rate, and financial leverage in each place, according to Wahed^26^. Hess et al.^27^ found the most potential solar heat absorption spots and ranked them in order of possible economic and energetic benefits based on the heat and mass balance of a sample sugar mill with few energy efficiency measures. Aktekeli et al.^28^ designed a new bi-fluid based photovoltaic thermal (PVT)-assisted heat pump dryer system with a payback period of 3.17 years. Aktas et al.^29^ determined the payback period of the PVT system to be 6.1 years by numerical and experimental analysis of a single-body PVT employing a variable air volume control technique.

Based on our review, Raja et al.^30^, Zaboli et al.^31^, Huminic and Huminic^32^, and Sharma et al.^33^, there was no actual research done to demonstrate how pulsing affected the solar collector’s performance. The exergy of solar collectors with pulsating flow hadn’t been studied before. It is worth noting that many recent studies have addressed this important topic, but have not discussed the pulsating flow^34–36^. Hence, The current project is centred on researching the output exergy of pulsating flow compared with continuous flow in the solar system. Companies always ask about the economic benefits of any technology, so the current work studies the economic output of pulsating flow. Governments and local communities are always looking for the environmental impact of any new technology being developed. Hence, we have conducted a detailed environmental study to clarify the impact of using pulsating flow on the performance of solar heaters.

## Experimental work

**Fig. 1:**
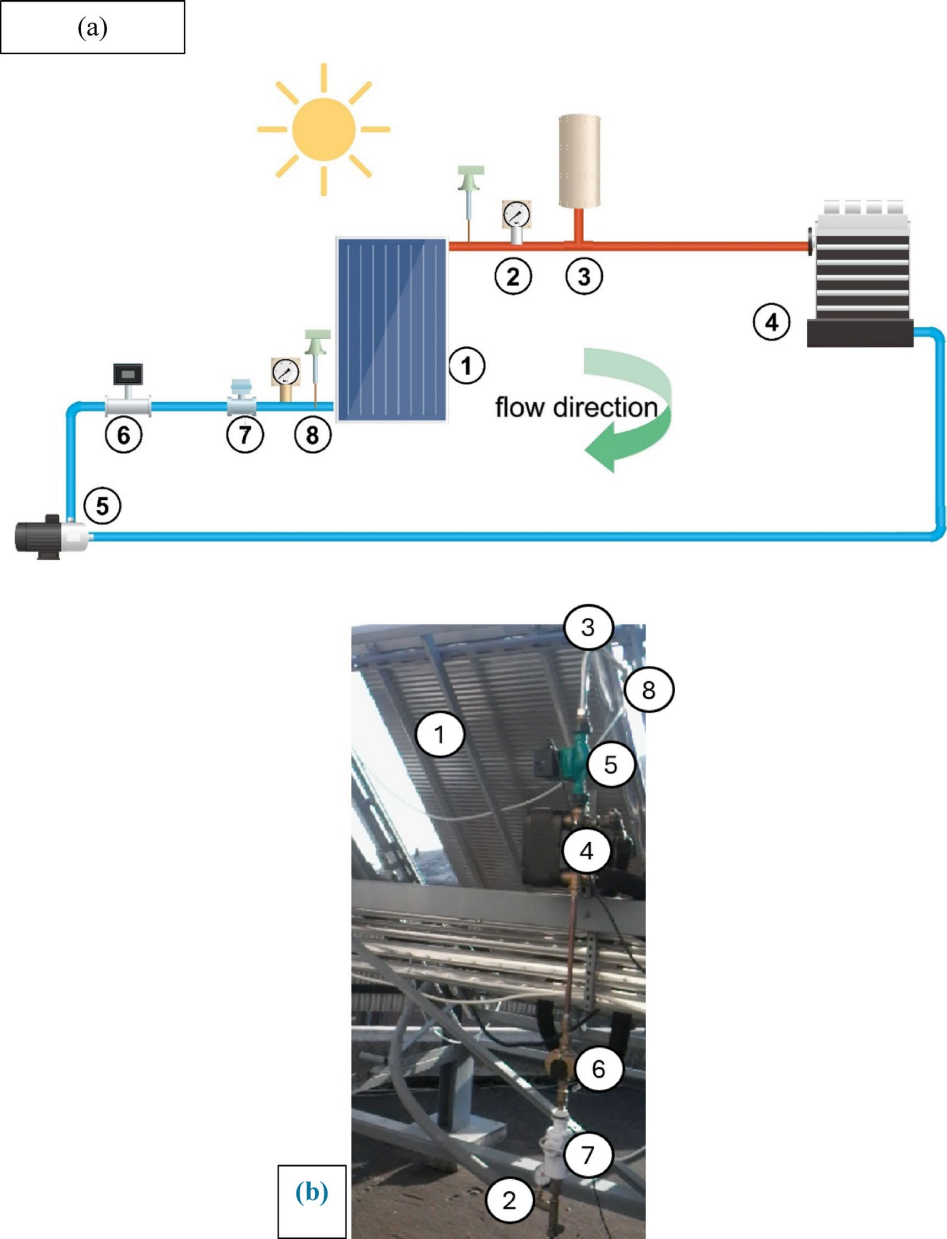
Layout for the used system: **a**) schematic drawing, **b**) real photo.

The flat plate kind of solar collector is the one that is used. An absorber plate collects solar radiation, which is then sent to the tube, where a pump circulates water. The movement of solar heat energy from the main loop to the other loop is facilitated by a heat exchanger. The secondary loop, a cooling device, receives the heat energy that has been gathered. Since the cycle is closed, the water is put back into the solar collector. Pulsating is produced by a solenoid valve in the system. It operates as electronically activated or deactivated and is utilised for regulating units that prevent flow or enable it. The solenoid valve was fitted before the inlet to the collector. An accumulator is placed after the collector to reduce pulsing and eliminate its impact on other system components. Figures 1 illustrates the general design of the system. At both the intake and the outflow, thermometers were used to measure the fluid’s temperature. The rate of fluid movement is measured by a flow meter. Table 1 shows the main specifications of measurement equipment.

The collector’s input and exit pressure differences are detected by employing a pressure transducer device. To collect measurement data, a data capture system is used. The weather station in the solar energy lab has a thermometer to measure the outdoor temperature and a sun radiation meter. Measurements were conducted between 10 a.m. and 3 p.m. Every 15 min, readings are obtained until the system stabilizes. The tests were conducted over days with almost identical meteorological circumstances.

(1. solar collector, 2. Pressure gauge, 3. Accumulator, 4. Heat exchanger, 5. pump, 6. flow meter, 7. Solenoid valve, 8. Thermocouple)

**Table 1:** Specifications of the equipment used in the experiments.

Instruments	Specification	Accuracy	Quantity
Thermocouple	K-type, 2 × 0.5 mm, −70 to250 $^\circ C$	± 0.5 $^\circ C$	3
Solar meter	0–1999 W/m^2^, operating temperature: 10–60 $^\circ C$	± 5%	1
Flow meter	100 to 700 L/hr.	± 10%	1
Pressure gauge	0–100 PSI operating temperature: −20–70 $^\circ C$	1.0%	1
Solenoid valve	Voltage110V AC flow coefficient of 4.5 Operational temperature range: −10 to 80 °C operate pressure 0 to 143 Psi	voltage range ± 10%	1

## Equations

The following equations are used to calculate exergy during the current work, as Amar et al.^37^, Mukherjee et al.^38^ Parsa et al.^39^ and Yanbolagh et al.^40^ as follows.

The energy and exergy of solar energy available at the inlet of the solar system

1 $$\:{\stackrel{\prime }{E}}_{{n}_{out}}=\stackrel{\prime }{m}{C}_{p}{\Delta\:}T$$

2 $$\:{\stackrel{\prime }{E}}_{\text{dest}}={\stackrel{\prime }{E}}_{\text{heat}}+{\stackrel{\prime }{E}}_{{m}_{in}}-{\stackrel{\prime }{E}}_{{m}_{out}}-{\stackrel{\prime }{E}}_{\text{work}}\:\:$$

3 $$\:\sum\:\:\left(1-\frac{{T}_{a}}{{T}_{\text{s}\text{}}}\right){\stackrel{\prime }{Q}}_{s}-\stackrel{\prime }{m}{\Delta\:}h-{T}_{a}{\Delta\:}s={\stackrel{\prime }{E}}_{\text{dest}}$$

4 $$\:{\stackrel{\prime }{Q}}_{s}={G}_{t}\left(\tau\:\alpha\:\right){A}_{c}$$

And for entropy changes, following Eq. (5) is used

5 $$\:{\Delta\:}s=\left({s}_{\text{out}}-{s}_{\text{in}}\right)=\stackrel{\prime }{m}{C}_{p}\text{ln}\left(\frac{{T}_{\text{out}}}{{T}_{\text{in}}}\right)-R\text{I}\text{n}\frac{{P}_{\text{out}}}{{P}_{\text{in}}}$$

Putting the values of terms from Eqs. (3–5) in Eqs. (2),

6 $$\:{\stackrel{\prime }{E}}_{\text{dest}}=\left(1-\frac{{T}_{a}}{{T}_{s}}\right){G}_{t}\left(\tau\:\alpha\:\right){A}_{c}-\stackrel{\prime }{m}{C}_{p}{\Delta\:}T+\stackrel{\prime }{m}\cdot\:{T}_{a}\left[{C}_{p}\text{ln}\left(\frac{{T}_{\text{out}}}{{T}_{\text{in}}}\right)-R\cdot\:{T}_{a}\text{ln}\left(\frac{{P}_{\text{out}}}{{P}_{\text{in}}}\right)\right]$$

The rate of exergy loss and exergy efficiency are given by Eq. (7) and Eq. (8)

7 $$\:{\stackrel{\prime }{E}}_{\text{dest}}={S}_{\text{gen}}^{\text{*}}\cdot\:{T}_{a}\:\:\:$$

8 $$\:{\eta\:}_{e}=1-\frac{{S}_{\text{gen}}^{\text{*}}\cdot\:{T}_{a}}{\left[1-\frac{{T}_{a}}{{T}_{\text{s}}}\right]{\stackrel{\prime }{Q}}_{s}}$$

Several papers are used to make economic and environmental analyses, such as Mukherjee et al.^38^, Essa^41^, Alqsair et al.^42^.

The capital recovery factor of the designed system can be calculated based on Eq. (9)

9 $$\:FCR=\frac{{i(1+i)}^{n}}{{(1+i)}^{n}-1}$$

Where $\:i$ is the interest rate, $\:n$ is the life span of the collector.

The First annual cost of solar systems is given in Eq. (10)

10 $$\:FACS=total\:cost\:of\:system*FCR$$

The annual maintenance cost of the used solar system is given in Eq. (11)

11 $$\:CAM=0.15\text{*}FACS$$

The overall salvage value of the system is calculated as follows:

12 $$\:S=0.1*total\:cost\:of\:system$$

Equation (10) shows the sinking fund factor.

13 $$\:SIFF=\frac{i}{{(1+i)}^{n}-1}$$

The yearly salvage value of the solar thermal system can be calculated as shown in Eq. (11)

14 $$\:ASVS=S*SIFF$$

The uniform annual cost of the current system is estimated with Eq. (12)

15 $$\:UACS=FACS+CAM-ASVS$$

The price of produced heat by the designed system is determined by the following Eq. (16)

16 $$\:PPHS=\frac{UACS}{{En}_{out}}$$

The calculation of exergoeconomic using the energy concept is as follows:

17 $$\:{\text{K}}_{\text{E}\text{X}\text{G}\text{O}}\left(\text{e}\text{n}\text{e}\text{r}\text{g}\text{y}\right)=\frac{\:{En}_{out}}{UACS}$$

The determination of exergoeconomic with the exergy concept is as follows:

18 $$\:{\text{K}}_{\text{E}\text{X}\text{G}\text{O}}\left(\text{e}\text{x}\text{e}\text{r}\text{g}\text{y}\right)=\frac{\:{EX\:}_{out}}{UACS}$$

The equation used to calculate greenhouse gas emissions is as follows:

19 $$\:net\:CO_2\:mitigation\:emission\:\left(tons\right)\:=\:\frac{\left(\right({En}_{out}\:\times\:n)\:-\:{E}_{in})\:\times\:\:1.58)}{1000}$$

20 $$\:Environeconomic\:parameter\:CO_2\:\left(\frac{\$}{year}\right)=net\:CO2\:emission*carbon\:credit$$

$$\:carbon\:credit=14.5\:\left(\frac{\$}{ton}\right)$$

21 $$\:net\:SO_2\:mitigation\:emission\:\left(tons\right)\:=\:\frac{\left(\right({En}_{out}\:\times\:n)\:-\:{E}_{in})\:\times\:\:0.012)}{1000}$$

22 $$\:net\:NO\:mitigation\:emission\:\left(tons\right)\:=\:\frac{\left(\right({En}_{out}\:\times\:n)\:-\:{E}_{in})\:\times\:\:0.046)}{1000}$$

The energy payback time based on energy is calculated as:

23 $$\:EPBT\:en=\frac{Total\:calculated\:Embodied\:energy}{\:{En}_{out}\:annual}$$

The energy payback time based on exergy is determined as

24 $$\:EPBT\:ex\:=\frac{Total\:calculated\:Embodied\:energy}{\:{EX}_{out}annual}$$

The energy production factor based on energy can be expressed as

25 $$\:EPF\:en=\:\frac{{En}_{out}annual}{Total\:calculated\:Embodied\:energy}=\frac{1}{EPBT\:en}$$

The energy production factor based on exergy is given as

26 $$\:EPF\:ex=\:\frac{{EX}_{out}annual}{Total\:calculated\:Embodied\:energy}=\frac{1}{EPBT\:ex}\:$$

**Table 2:** The design and analysis parameters of the proposed system.

Collector parameters	Value
Type	Black paint header-riser flat plate
Glazing	Double glass
Agent fluid in flow ducts	Water
Adhesive resistance,	Negligible
Wind speed,	3.5 m/s
Collector tilt	30°
Apparent sun temperature, Ts	4350 K ^43^
Plate thickness,	0.002 m
Effective product transmittance-absorptance or optical efficiency,	0.9
Emissivity of the absorber plate,	0.91
Emissivity of the covers,	0.89
Glass covers’ distance	0.04 m
Thickness of the back insulation	0.08 m
Thickness of the sides’ insulation	0.04 m
Thermal conductivity of the absorber plate,	384 W/m K
Thermal conductivity of the insulation,	0.05 W/m K
Tubes’ centre-to-centre distance,	0.1 m
Inner diameter of pipes,	0.019 m

## Uncertainty analysis

Uncertainty analysis is used to illustrate the accuracy of the measurements for the present investigation. The results of the uncertainty analysis for the solar collector efficiency are presented in this section of the study. Can et al.^44^ described the more accurate approach to quantifying experimental uncertainty. The approach is based on a detailed description of the uncertainties associated with the basic results of experiments. Assume that functions are assigned to the independent variables x_1, x_2, x_3,…., x_n, and R. Consequently, R = R(x_1,x_2,x_3,….,x_n). Let u_R stand for the uncertainty of the outcome, and let u_1, u_2, u_3,…., u_n stand for the uncertainties in the independent variables. The following Eq. (27) is a statement of the outcome’s uncertainty:

27 $$\:{u}_{R}=\sqrt{{\left(\frac{\partial\:R}{\partial\:{x}_{1}}{u}_{1}\right)}^{2}+{\left(\frac{\partial\:R}{\partial\:{x}_{2}}{u}_{2}\right)}^{2}+\dots\:+{\left(\frac{\partial\:R}{\partial\:{x}_{n}}{u}_{n}\right)}^{2}}$$

For every experimental test, the highest thermal efficiency uncertainties are approximately 3.5%.

## Results

In this part, the results are shown. Results for exergy, economic, exergyecomic, environmental, and energy matrices are discussed in detail in the following parts.

### Exergy results

**Fig. 2:**
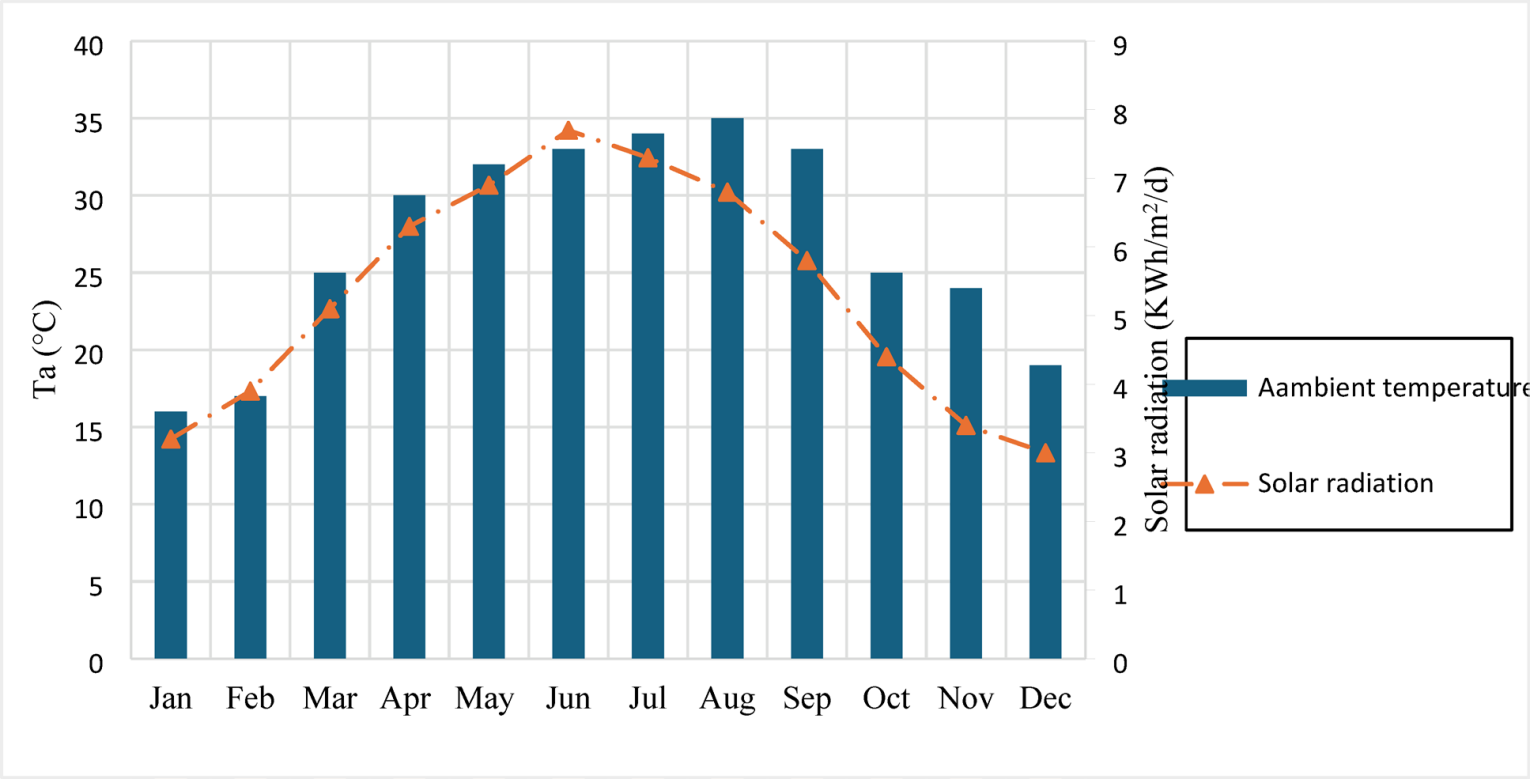
Average ambient temperature and average solar radiation for each month of the year.

Applying an exergy assessment to a solar collector guide to reduce exergy losses and aid designers in creating the best possible design. One of the two approaches to examining the second law is the idea; the other is the development of entropy from irreversibility. Nonetheless, the outcomes of both approaches are essentially the same. From the perspective of optimising exergy efficiency, it is possible to reduce the exergy destroyed during thermodynamic operations. It depends on solar radiation and the sun’s apparent temperature. Table 2 shows the main design and analysis parameters of the proposed system. Figure 2 shows the Average ambient temperature and average solar radiation for each month of the year. For a solar system to be evaluated for exergetic performance, its entropy generation and exergy destruction (Exdest) must be measured. Entropy generation is regarded as an indicator of irreversibility because irreversible losses affect the functioning of the solar system, resulting in rising entropy and falling thermal efficiency. The energy and exergy analysis of the used system was calculated based on Eqs. (1–9). The average monthly energy and exergy of the system are collected in Tables 3 and 4, respectively. Exergy destruction, entropy generation, and exergy efficiency as monthly values are collected in Tables 5 and 6, and 7, respectively.

**Table 3:** Monthly energy production (kWh/m^2^).

	Continuous flow			Pulsating flow 10 Hz			Pulsating flow 6 Hz			Pulsating flow 4 Hz		
	360 L/hr	420 L/hr	480 L/hr	360 L/hr	420 L/hr	480 L/hr	360 L/hr	420 L/hr	480 L/hr	360 L/hr	420 L/hr	480 L/hr
Jan	0.2	0.3	0.3	0.4	0.4	0.4	0.7	0.9	1.0	1.0	1.3	1.3
Feb	0.8	1.0	1.5	1.0	1.2	1.7	2.0	2.4	2.6	2.4	2.9	3.0
Mar	13.1	13.3	14.0	14.3	14.9	15.3	16.8	17.7	18.9	17.1	20.8	21.3
Apr	32.0	32.5	33.0	34.9	33.3	34.0	38.8	40.1	42.9	39.2	43.0	44.1
May	60.1	64.1	65.0	63.4	72.9	73.8	73.5	75.7	78.9	74.5	80.5	81.4
Jun	79.2	82.1	84.0	80.2	91.1	95.6	93.5	95.3	100.2	94.9	101.4	102.4
Jul	84.4	89.1	91.6	90.6	97.3	102.1	98.3	100.4	106.9	104.5	108.1	109.6
Aug	72.6	74.2	75.0	74.0	83.4	88.2	83.4	86.3	90.4	87.2	90.6	91.0
Sep	48.5	49.0	49.3	49.6	52.3	56.4	54.2	54.7	57.1	57.3	60.4	61.1
Oct	14.5	14.7	14.8	15.0	16.5	17.5	19.4	20.2	21.8	21.0	22.2	22.6
Nov	0.7	0.7	0.8	1.4	1.9	2.1	1.6	2.3	2.8	1.7	2.7	3.0
Dec	0.2	0.2	0.3	0.3	0.7	1.2	0.6	1.0	1.4	0.8	1.4	1.6

**Table 4:** Monthly useful exergy(kWh/m^2^).

	Continuous flow			Pulsating flow 10 Hz			Pulsating flow 6 Hz			Pulsating flow 4 Hz		
	360 L/hr	420 L/hr	480 L/hr	360 L/hr	420 L/hr	480 L/hr	360 L/hr	420 L/hr	480 L/hr	360 L/hr	420 L/hr	480 L/hr
Jan	0.06	0.05	0.04	0.08	0.07	0.07	0.18	0.15	0.12	0.23	0.21	0.16
Feb	0.31	0.18	0.15	0.32	0.20	0.18	0.47	0.40	0.33	0.54	0.46	0.39
Mar	2.88	2.38	2.38	2.88	2.50	2.50	3.42	2.95	2.78	3.82	3.32	2.78
Apr	6.79	5.82	5.80	6.89	6.29	6.09	7.75	6.67	6.41	7.90	6.85	6.48
May	13.37	11.46	10.89	13.81	12.22	11.06	14.25	12.59	12.13	14.58	12.82	11.98
Jun	17.27	14.68	14.35	17.88	15.26	14.20	18.10	15.84	15.43	18.34	16.14	15.26
Jul	18.83	15.93	15.29	19.09	16.30	15.79	19.30	16.68	16.21	19.62	17.20	16.80
Aug	15.42	13.26	13.15	16.49	14.74	13.29	16.52	14.34	13.75	16.48	14.41	14.01
Sep	10.14	8.76	8.79	10.55	9.25	8.81	10.80	9.09	8.94	10.94	9.61	9.21
Oct	3.05	2.63	2.63	3.28	2.92	2.62	3.95	3.36	3.21	4.06	3.54	3.38
Nov	0.16	0.13	0.13	0.39	0.34	0.24	0.51	0.38	0.26	0.54	0.43	0.27
Dec	0.06	0.04	0.04	0.23	0.12	0.05	0.25	0.17	0.10	0.29	0.22	0.13

**Table 5:** Monthly exergy destruction for continuous and pulsating flow (kWh/m^2^).

	Continuous flow			Pulsating flow 10 Hz			Pulsating flow 6 Hz			Pulsating flow 4 Hz		
	360 L/hr	420 L/hr	480 L/hr	360 L/hr	420 L/hr	480 L/hr	360 L/hr	420 L/hr	480 L/hr	360 L/hr	420 L/hr	480 L/hr
Jan	89.56	89.57	89.59	89.55	89.55	89.55	89.44	89.47	89.51	89.39	89.41	89.46
Feb	101.61	101.74	101.77	101.60	101.72	101.74	101.45	101.52	101.59	101.38	101.46	101.53
Mar	144.38	144.89	144.89	144.39	144.77	144.77	143.85	144.32	144.49	143.45	143.95	144.49
Apr	169.04	170.02	170.03	168.94	169.55	169.75	168.08	169.17	169.43	167.93	168.99	169.35
May	185.53	187.44	188.01	185.10	186.69	187.85	184.65	186.32	186.77	184.32	186.09	186.92
Jun	197.48	200.07	200.40	196.87	199.49	200.55	196.65	198.91	199.32	196.41	198.61	199.49
Jul	191.50	194.40	195.04	191.24	194.03	194.54	191.02	193.65	194.11	190.71	193.13	193.53
Aug	180.46	182.61	182.72	179.39	181.13	182.59	179.36	181.54	182.12	179.39	181.46	181.86
Sep	151.62	153.00	152.97	151.21	152.51	152.95	150.96	152.67	152.82	150.82	152.15	152.55
Oct	124.01	124.42	124.42	123.78	124.13	124.44	123.11	123.69	123.85	123.00	123.52	123.67
Nov	94.87	94.91	94.91	94.64	94.70	94.79	94.53	94.65	94.77	94.50	94.61	94.76
Dec	86.70	86.72	86.72	86.53	86.63	86.70	86.50	86.59	86.66	86.47	86.53	86.63

**Table 6:** Monthly entropy generation for continuous and pulsating flow(kW/km^2^).

	Continuous flow			Pulsating flow 10 Hz			Pulsating flow 6 Hz			Pulsating flow 4 Hz		
	360 L/hr	420 L/hr	480 L/hr	360 L/hr	420 L/hr	480 L/hr	360 L/hr	420 L/hr	480 L/hr	360 L/hr	420 L/hr	480 L/hr
Jan	0.310	0.310	0.310	0.310	0.310	0.310	0.309	0.310	0.310	0.309	0.309	0.310
Feb	0.350	0.351	0.351	0.350	0.351	0.351	0.350	0.350	0.350	0.350	0.350	0.350
Mar	0.485	0.486	0.486	0.485	0.486	0.486	0.483	0.484	0.485	0.481	0.483	0.485
Apr	0.558	0.561	0.561	0.558	0.560	0.560	0.555	0.558	0.559	0.554	0.558	0.559
May	0.608	0.615	0.616	0.607	0.612	0.616	0.605	0.611	0.612	0.604	0.610	0.613
Jun	0.645	0.654	0.655	0.643	0.652	0.655	0.643	0.650	0.651	0.642	0.649	0.652
Jul	0.624	0.633	0.635	0.623	0.632	0.634	0.622	0.631	0.632	0.621	0.629	0.630
Aug	0.586	0.593	0.593	0.582	0.588	0.593	0.582	0.589	0.591	0.582	0.589	0.590
Sep	0.495	0.500	0.500	0.494	0.498	0.500	0.493	0.499	0.499	0.493	0.497	0.499
Oct	0.416	0.418	0.418	0.415	0.417	0.418	0.413	0.415	0.416	0.413	0.414	0.415
Nov	0.319	0.320	0.320	0.319	0.319	0.319	0.318	0.319	0.319	0.318	0.319	0.319
Dec	0.297	0.297	0.297	0.296	0.297	0.297	0.296	0.297	0.297	0.296	0.296	0.297

**Table 7:** Monthly exergy efficiency for continuous and pulsating flow.

	Continuous flow			Pulsating flow 10 Hz			Pulsating flow 6 Hz			Pulsating flow 4 Hz		
	360 L/hr	420 L/hr	480 L/hr	360 L/hr	420 L/hr	480 L/hr	360 L/hr	420 L/hr	480 L/hr	360 L/hr	420 L/hr	480 L/hr
Jan	0.07%	0.06%	0.04%	0.08%	0.08%	0.08%	0.20%	0.17%	0.13%	0.26%	0.23%	0.18%
Feb	0.30%	0.18%	0.14%	0.31%	0.20%	0.17%	0.46%	0.39%	0.33%	0.53%	0.45%	0.38%
Mar	1.96%	1.62%	1.62%	1.96%	1.70%	1.70%	2.32%	2.00%	1.89%	2.60%	2.25%	1.89%
Apr	3.86%	3.31%	3.30%	3.92%	3.57%	3.46%	4.41%	3.79%	3.64%	4.49%	3.90%	3.69%
May	6.72%	5.76%	5.48%	6.94%	6.14%	5.56%	7.17%	6.33%	6.10%	7.33%	6.44%	6.02%
Jun	8.04%	6.84%	6.68%	8.33%	7.11%	6.61%	8.43%	7.38%	7.18%	8.54%	7.52%	7.10%
Jul	8.95%	7.57%	7.27%	9.08%	7.75%	7.51%	9.18%	7.93%	7.71%	9.33%	8.18%	7.99%
Aug	7.87%	6.77%	6.71%	8.42%	7.53%	6.78%	8.43%	7.32%	7.02%	8.42%	7.36%	7.15%
Sep	6.27%	5.42%	5.43%	6.52%	5.72%	5.45%	6.68%	5.62%	5.53%	6.76%	5.94%	5.69%
Oct	2.40%	2.07%	2.07%	2.58%	2.30%	2.06%	3.11%	2.65%	2.52%	3.19%	2.79%	2.66%
Nov	0.17%	0.13%	0.13%	0.41%	0.35%	0.26%	0.53%	0.40%	0.28%	0.57%	0.45%	0.29%
Dec	0.07%	0.04%	0.04%	0.26%	0.14%	0.06%	0.29%	0.19%	0.11%	0.33%	0.26%	0.15%

Exergy efficiency is one of the main parameters to evaluate the performance of the solar collector with any modification. It can be calculated based on Eq. (8). It is the ratio between the useful exergy and solar exergy. Table 7 contains the monthly exergy efficiency for continuous and pulsating flow at different flow rates. Exergy efficiency increases from 0.04% to 9.33% for continuous and pulsating flow. The results find that the exergy efficiency decreases with the rise in flow rate. The pulsating flow has a higher exergy efficiency compared with continuous flow. The lower frequency indicated higher exergy efficiency.

The exergy analysis of a solar collector primarily depends on key operating parameters such as flow rate, outlet temperature, and available solar radiation. At lower flow rates, the exergy efficiency tends to increase due to a reduction in entropy generation and exergy destruction^37^. This occurs because lower flow rates allow greater heat absorption and higher outlet fluid temperatures, which in turn minimize entropy generation and enhance exergy performance.

In the present study, the investigated flow rates correspond to turbulent Reynolds numbers in the range of 6700–9000. Under pulsatile flow conditions, turbulence within the buffer layer near the wall is generated by motions comparable to those in steady turbulent flow; however, it subsequently decays and propagates radially outward in a monotonic fashion. The fluid in pulsatile motion experiences continuous acceleration and deceleration, resulting in a flow pattern analogous to steady flow, where inertial and centrifugal forces are more uniformly distributed. During the deceleration phase, as centrifugal effects become dominant, vortices expand across the pipe cross-section and gradually migrate toward the pipe centerline. Conversely, during peak acceleration, secondary flow structures become weak or nearly absent.

A comparison between acceleration and deceleration phases at maximum velocity reveals that velocity fluctuations are more pronounced during deceleration. This phenomenon can be attributed to re-laminarization at low flow velocities, where turbulence intensity diminishes as the flow transitions from turbulent to laminar. Nevertheless, during deceleration, residual turbulent structures persist, delaying the re-laminarization process and leading to increased velocity fluctuations compared to the acceleration phase. These observations are consistent with previous findings reported in the literature^45–48^.

The observed increase in outlet temperature in pulsating flow conditions arises from enhanced turbulence, vibration, and mixing induced by the periodic velocity variations, particularly at low pulsation frequencies. During portions of the pulsation cycle when the fluid remains momentarily stagnant, it is exposed to solar radiation for a longer duration, thereby absorbing more heat. Consequently, this mechanism enhances the outlet temperature, reduces heat losses, decreases entropy generation, and minimizes exergy destruction. The enhancement in outlet temperature and, therefore, exergy efficiency is most significant under low-frequency pulsating flow conditions. Furthermore, under high solar radiation levels, such as those occurring during summer, the available thermal energy increases, leading to higher exergy efficiency and improved overall performance of the solar collector system.

### Economic results

The purpose of economic evaluation is to elucidate the potential of solar thermal energy as a clean energy source. Governments, businesses, and individuals will not spend on solar thermal energy unless they are aware of its financial benefits. Therefore, the present study presents a discussion of economic parameters in the case of utilising solar thermal energy. The impact on ecosystems of various kinds of fluid flow is demonstrated. Both pulsating and continuous flow are explained. The effect of pulsation frequency is discussed. An economic analysis based on the uniform annual cost of systems (UASC) is conducted. In every case under study, several parameters are established, including the system’s overall cost and lifespan, which is set at 20 years. The interest rate is about 10%. Calculations are made to calculate the following factors: sinking fund factor (SIFF) and the capital recovery factor (FCR). Costs such as the first annual costs of systems (FACS) and the cost of annual systems maintenance (CAM) are estimated. The overall salvage value of systems (S) is expected. The price of heat produced by systems (PPHS) is estimated based on the annual average energy produced (En_out_). The annual average energy produced was given from the previous work of.

The exergoeconomic results for the solar collector based on the energy/exergy concept are displayed. The environmental variable values are collected. It is calculated how much CO₂, SO₂, and NO emissions are reduced when solar energy is combined with pulsating flow. The energy matrix relationships based on the energy/exergy concept are compiled. The calculation of energy payback time (EPBT) and EPF is based on the energy and exergy concepts.

#### Uniform annual cost results

**Fig. 3:**
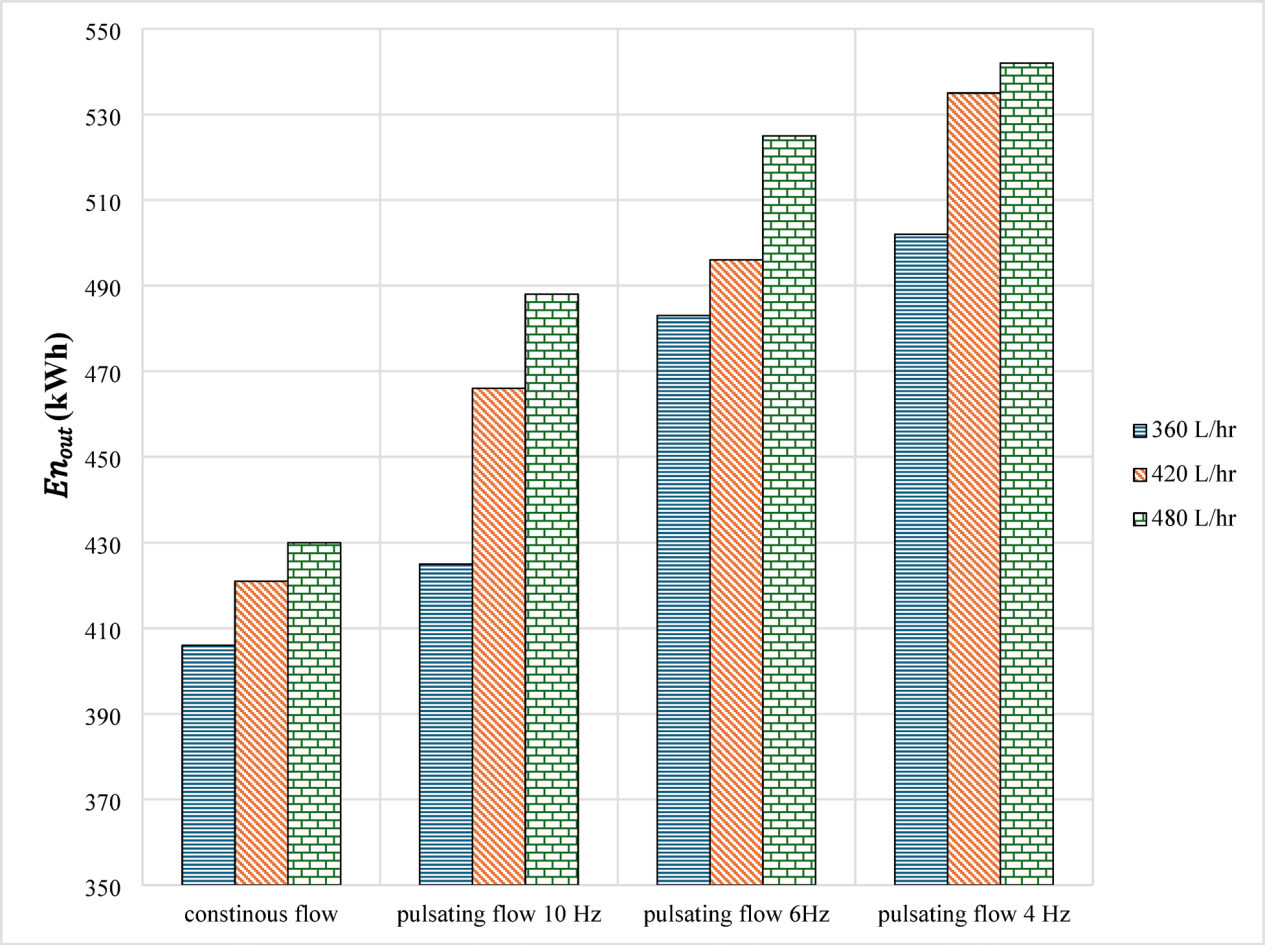
Average annual energy production for continuous and pulsating flow.

**Fig. 4:**
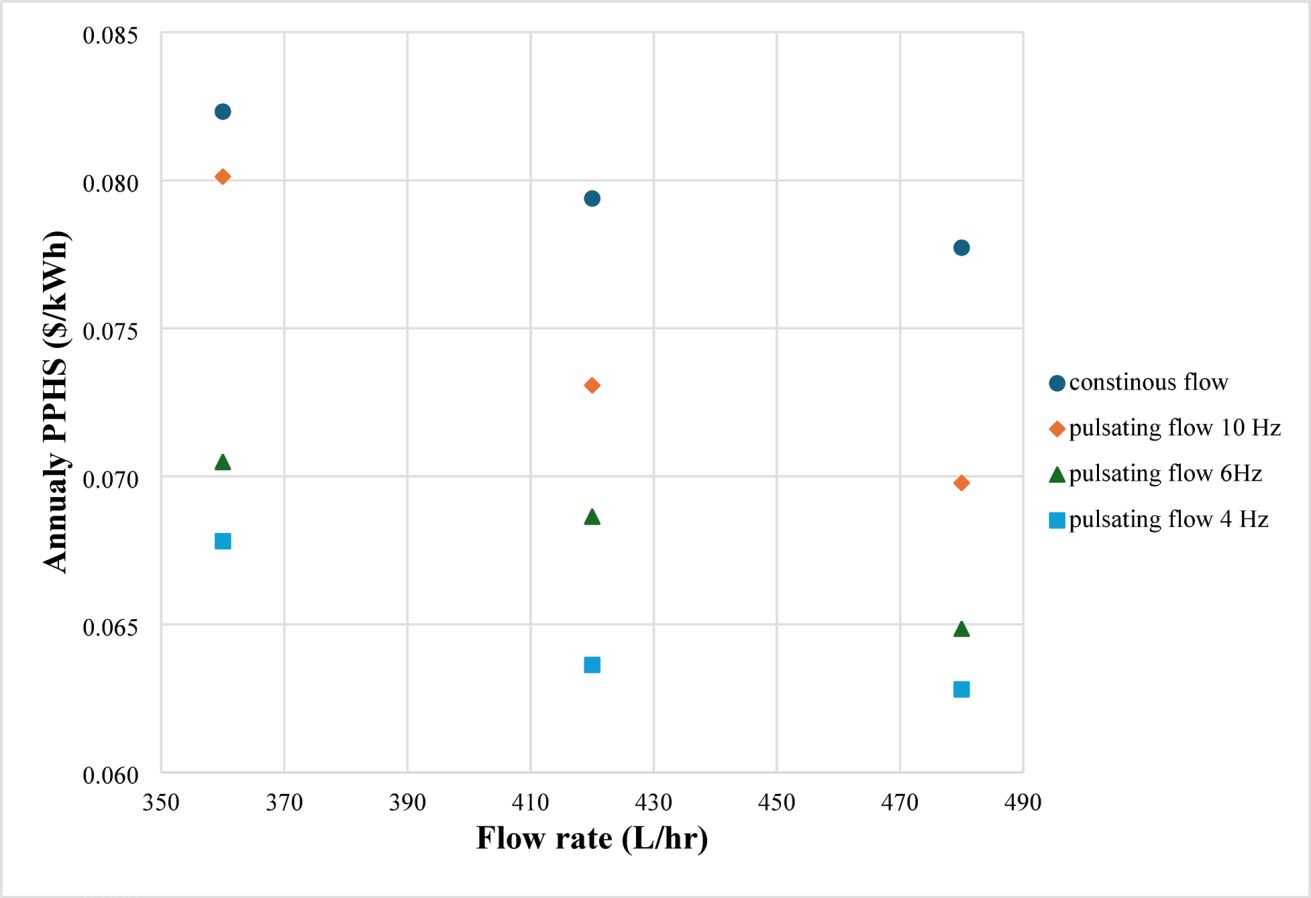
Average annual price of the produced heat by systems for continuous and pulsating flow.

Economic analysis is one of the most crucial elements that needs to be evaluated for any energy framework. To investigate the system’s effectiveness from an economic standpoint, a study employing the uniform annual cost (UAC) technique is currently underway. In addition to the cost of the nanoparticles and electricity, the total cost included the cost of the system components (flat plate collector, copper tubes, the cooling unit, solenoid valves, and measurement tools). 10% was chosen as the interest rate. Twenty years was selected as the system’s lifespan. The primary result of economic analysis is the cost of the heat that systems produce. The yearly price of each kW of energy generated by the system is referred to here. Data for economic analysis based on the uniform annual cost of systems is given in Table 8. Figures 3 shows the annual amount of thermal energy produced by the solar collector in the case of continuous and pulsating flow. The effect of pulsating frequency is presented. Figures 4 expresses the average annual price of the heat produced by solar thermal systems for continuous and pulsating flow based on the uniform annual cost techniques.

Based on data collected, the solar collector system, including all accessories (tubes, pump, heat exchanger, etc.), costs around $250. The cost of electricity needed to circulate the pump is $0.661, according to the Egyptian electricity cost system. That type of consuming energy is added to all cases of pulsating and continuous flow. For pulsating flow only, the price of the solenoid valve is added. The price of energy needed to activate the valve differs according to the valve frequency. Based on the life cycle of the solar collector system, which is 20 years, and the interest rate, which is 10%, the capital recovery factor is calculated to be 0.1175. It applies a predetermined discount rate to the present value and transforms it into a sequence of equal yearly cash flows over the project’s duration. It can be calculated based on Eq. (9). The first annual costs of systems (FACS) are calculated as given in Eq. (10). It reflects the recovery money, which is given as the base cost of the collector system. The yearly maintenance cost (CAM) of the solar system is calculated by Eq. (11). It is taken as 15% of the first annual cost. Overall, salvage value (S) is the amount that solar system equipment is worth after its intended lifespan is over. In other words, when subtracting the machinery’s depreciation from its initial cost, the salvage value is given. In the current work, it is 10% of the total system cost. It is presented in Eq. (12). Usually, the SIFF is used to calculate the amount that needs to be saved every period to cover a future financial obligation, it calculated to be 0.01746 for the current work. It is calculated utilising equations (13). It is calculated based on the life span of the system and interest rate, which is equal to 0.01746 in the current results.

The annual salvage value of systems (ASVS) is computed as applied in Eq. (14). The uniform annual cost (UAC) is the main output of the method. It is estimated as in Eq. (15). It is the sum of the first annual cost plus the annual maintenance cost, subtracted from the yearly salvage values of the solar system. The annual average heat energy produced by the solar system is presented in Table 8.

Equation (16) shows the method to calculate the price of the heat energy produced by the solar system. It is the ratio between the uniform annual cost and the annual average heat energy produced (PPHS) by the solar system. It is the method to clarify the cost of energy, which is the main concern of the current work. It is a good way to compare the different methods to enhance energy production by the solar collector.

**Table 8:** The uniform annual cost of solar system, continuous and pulsating flow (UASC).

Case	Flow rate (L/h)	total cost of the system	FACS($)	CAM($)	S($)	ASVS($)	UACS($)	Enout (KW h)	PPHS $/kWh/year
**Continuous flow**	360	250.66	29.44	4.42	25.07	0.44	33.42	406	0.082
	420	250.66	29.44	4.42	25.07	0.44	33.42	421	0.079
	480	250.66	29.44	4.42	25.07	0.44	33.42	430	0.078
**Pulsating flow 10 Hz**	360	255.39	30.00	4.50	25.54	0.45	34.05	425	0.080
	420	255.39	30.00	4.50	25.54	0.45	34.05	466	0.073
	480	255.39	30.00	4.50	25.54	0.45	34.05	488	0.070
**Pulsating flow 6 Hz**	360	255.34	29.99	4.50	25.53	0.45	34.05	483	0.070
	420	255.34	29.99	4.50	25.53	0.45	34.05	496	0.069
	480	255.34	29.99	4.50	25.53	0.45	34.05	525	0.065
**Pulsating flow 4 Hz**	360	255.31	29.99	4.50	25.53	0.45	34.04	502	0.068
	420	255.31	29.99	4.50	25.53	0.45	34.04	535	0.064
	480	255.31	29.99	4.50	25.53	0.45	34.04	542	0.063

Solar energy production is the main output from the collector. Figures 3 shows the average annual energy production for continuous and pulsating flow. It was found that the average annual solar energy in the case of continuous flow increased from 406 to 430 kWh for all flow rates. When the pulsating flow with 10 Hz was applied, these values became higher. Although with a lower frequency of 6 Hz, values were 483,496, and 525 kWh/m² for flow rates of 360, 420, and 480 L/hr, respectively. The lowest frequencies were 4 Hz, and their values ranged from 502 to 542 kWh. Based on the given results, it was found that pulsating flow gives more energy production compared with continuous flow. Also, using lower values of frequency has a positive impact on generating more solar energy. That is because of fluctuation and turbulence in pulsating flow, which is higher compared with a continuous one. Also, with lower frequency, more mixing and collapsing between particles is found. It was proved that a higher flow rate absorbed more solar energy. The highest amount of energy in the current work was 542 kWh/m² for 4 Hz at a flow rate of 480 L/hr. Generally, it was calculated that energy production can be enhanced up to 33.5% using the current conditions.

The price of energy is an essential issue for customers and companies. They need enhanced techniques to reduce the cost of energy by increasing the amount of energy produced with a lower increase in added money. So, calculating the average heat energy produced by the system is a must. In the current work, the unit for average heat energy produced by the system is $/kWh, using the US dollar to make it easy to compare worldwide. Figures 4 represents the average annual price of the heat produced by systems for continuous and pulsating flow. The price of each kWh of solar energy was between 0.082 and 0.78 $. That cost dramatically decreased in the case of a pulsating flow of 10 Hz. With the lower frequency of 6 Hz, the smallest cost was achieved. The last price of energy was for the case of 4 Hz, as it was between $0.068 and $0.063 for different flow rates. It was noted that pulsating flow had a lower cost compared with a continuous one. Also, a higher flow rate decreased the cost. The lower the frequency used, the lower the price paid for energy. The response is the remarkable increase in energy produced in the case of low-frequency pulsating flow. Although a higher cost was detected for establishing the system with pulsating as a solenoid valve, and a calculator tank was added to the pulsating system, the increase in energy was higher. Simply, the currently studied cases reduced the energy price by 23.2%.

### Exergoeconomic results

**Table 9:** Exergoeconomic values based on the energy/exergy concept for continuous and pulsating flow.

Case	Flow rate (L/h)	UACS ($)	Enout (KW h)	EXout (KW h)	KEXGO, (energy) kWh/$	KEXGO, (exergy) kW h/$
**Continuous flow**	360	33.42	406	88.35	12.15	2.64
	420	33.42	421	75.32	12.60	2.25
	480	33.42	430	73.64	12.87	2.20
**Pulsating flow 10 Hz**	360	34.05	425	91.88	12.48	2.70
	420	34.05	466	80.20	13.68	2.36
	480	34.05	488	74.90	14.33	2.20
**Pulsating flow 6 Hz**	360	34.05	483	95.51	14.19	2.80
	420	34.05	496	82.62	14.57	2.43
	480	34.05	525	79.67	15.42	2.34
**Pulsating flow 4 Hz**	360	34.04	502	97.35	14.75	2.86
	420	34.04	535	85.22	15.72	2.50
	480	34.04	542	80.85	15.92	2.38

Exergoeconomics is a method that entails figuring out the concept of energy’s economic value to maximise the cost of processing. Applying the economic idea of energy cost, which is a thermodynamic property suitable for this use since it integrates the amount of energy with its quality factor, blends thermodynamic and economic analysis. The term “exergoeconomic” was first used by Tsatsaronis and Winhold^49^ to refer to the conjunction of economic and exergy assessment. This approach relies on the idea that cost should be allocated to energy because it is a logical way to put a financial value on the flow of energy and system inefficiencies. The irreversible annihilation of energy is the physical phenomenon that ties thermodynamics and economics together. Irreversibility is the metric by which we gauge the level of excellence of a part or an entire installation since it denotes the loss of energy’s utility because of its destruction, either within or outside of the system. The cost of the final product is directly proportional to the amount of natural resources used in the process, which increases with the irreversibility of the process. This enables us to understand how resources might be used more efficiently to reduce energy use^50^.

The calculation of exergoeconomic is carried out based on Eqs. (17) and (18). Equation (17) used the energy output from the solar system, while Eq. (18) relied on exergy output from the solar system. In Eq. (17), exergoeconomic (energy) is calculated as the ratio between the annual energy produced by the solar system and the uniform annual cost. In Eq. (18), exergoeconomic (exergy) is determined as the ratio between the annual exergy produced by the solar system and the uniform annual cost. The unit used in both exergoeconomic parameters is kWh/$. Results for exergoeconomic values are given in Table 9 besides the results of energy and exergy produced annually by the solar collector. Results for continuous flow are changed from 12.15 to 12.87 in the case of exergoeconomic (energy), while they move from 2.64 to 2.2 for exergoeconomic (exergy). The case of pulsating flow with 10 Hz expressed the following results: 12.48, 13.68, and 14.33 for exergoeconomic (energy) and 2.7, 2.36, and 2.2 for exergoeconomic (exergy) at all flow rates. Higher values are found when a 6 Hz pulsating flow is carried out. The maximum values are observed at 4 Hz. Based on the given results, it is found that exergoeconomic (energy) values are always higher than exergoeconomic (exergy) values. That is because the energy outlet from the system is more than the exergy. The exergoeconomic (energy) and exergoeconomic (exergy) for continuous flow are less than pulsating flow. Also, increasing pulsating flow has a negative effect on exergoeconomic (energy) and exergoeconomic (exergy). Although the pulsating flow consumes more money for establishment, operation, and maintenance, it relatively produces more energy. As a result, the final values for exergoeconomic (energy) and exergoeconomic (exergy) are higher in the case of pulsating. The maximum value for exergoeconomic (energy) is 15.92; for exergoeconomic (exergy), it is 2.86. The lowest values for exergoeconomic (energy) and exergoeconomic (exergy) are 12.15 and 2.2, respectively. The enhancement ratio of exergoeconomic (energy) is 31%.

### Environmental analysis

Environmental analysis is one of the main issues of the current work. The main aim of using solar energy is its positive impact on the environment. Many methods are used to clarify the environmental analysis of heat transfer machines. In the current work calculation, the saving of greenhouse gases when an enhanced solar system is used is the utilized technique. The amount of CO₂, SO₂, and NO gases that can be mitigated is calculated based on Eqs. 19, 21, and 22, respectively. The results for both continuous flow and pulsating flow are presented in Table 10. According to current results, 9.11, 9.59, and 9.87 tonnes of CO₂ can be saved in the case of using continuous flow. For pulsating flow with 10 Hz, 9.71, 11, and 11.7 tonnes of CO₂ migrated. More CO₂ can be saved with 10 Hz pulsating flow to be 11.54, 11.96, and 12.87 flow rates of 360, 420, and 480 L/hr, respectively. For 4 Hz pulsating flow, the results are 12.15, 13.19, and 13.41 tonnes of CO₂ for flow rates of 360, 420, and 480 L/hr, respectively. The SO₂ gases can be migrated from 0.07 tonnes to 0.1 tonnes with pulsating flow at the used flow rates. For NO gas values, no less than 0.27 tonnes and no more than 0.39 tonnes can be saved. The net CO₂ emission mitigation reached 13.4 tonnes/year for the pulsating flow of 4 Hz. The number of financial resources that may be saved by using solar energy systems to prevent one tonne of CO₂ emissions is known as the environmental parameter. It can be calculated as given in Eq. (20). Its unit is $/year. The carbon credit in the current study is taken as $14.5/tonne. Table 10 shows the environmental parameters. For continuous flow, the value is between $132 and $143 per year. The money is higher in the case of pulsating to 169.7 $/year for 10 Hz. A greater increase is noted for 6 Hz pulsating flow to 186.6 $ per year. The maximum yearly money is for a 4 Hz pulsating flow to be 176, 191, and 194 $ at flow rates of 360, 420, and 480 L/hr, respectively. Based on those results, pulsating flow saves the environment from greenhouse gases more than continuous flow. Lower frequency helps to gain more money from carbon environment parameters every year of the solar system’s working.

**Table 10:** The environmental analysis values for continuous and pulsating flow.

Case	Flow rate (L/h)	Net CO_2_ emission mitigation (ton CO_2_/year)	Net SO2 emission mitigation (ton SO_2_/year)	Net NO emission mitigation (ton NO/year)	Environeconomic parameter CO_2_ ($/year)
**Continuous flow**	360	9.12	0.07	0.27	132.17
	420	9.59	0.07	0.28	139.04
	480	9.87	0.07	0.29	143.17
**Pulsating flow 10 Hz**	360	9.71	0.07	0.28	140.79
	420	11.01	0.08	0.32	159.58
	480	11.70	0.09	0.34	169.66
**Pulsating flow 6 Hz**	360	11.54	0.09	0.34	167.40
	420	11.955	0.09	0.35	173.36
	480	12.87	0.10	0.37	186.64
**Pulsating flow 4 Hz**	360	12.146	0.09	0.35	176.12
	420	13.19	0.10	0.38	191.24
	480	13.41	0.10	0.39	194.45

### Energy-matrices

Embodied energy is the energy used when a material is being manufactured. All the materials used to build and maintain technical installations, as well as the energy used during operation, make up the solar energy system. The pump power consumption is added, and the operation power for the solenoid valve. The total embodied energy of the solar system with its operation is given in Table 11. By comparing the energy needed to make different building materials, the embodied energy concept can compare their sustainability. The project’s payback period is ascertained by contrasting the initial investment cost with the project’s yearly profits.

A company can calculate the time it requires for an investment to return the initial money required for creating it by comparing these values. Since the return on investment enables more expansion, a project with a faster return on investment appears more attractive. The time it requires for a power system to generate as much energy as it requires to be produced is known as Energy Payback Time, or EPBT. It is determined by dividing the total energy/exergy input by the energy/exergy of the yearly production. It can be calculated based on energy or exergy generated by the solar system. The energy payback time based on energy and exergy is calculated as given in Eqs. (23) and (24), respectively. It can also be calculated based on energy or exergy, as demonstrated in Eqs. (25) and (26), respectively. Results for energy-matrices value are shown in Table 12.

The embodied energy for a continuous flow system is the lowest case, with 2351 kWh. Those values increased for pulsating flow as the selenide valve consumed electrical power. It increased from 2352.47 kWh at 4 Hz to 2353.2 kWh at 6 Hz. The maximum value for 10 Hz is 2354.7, as the activation of the valve consumes much power in that case. The EPBT based on energy for the continuous flow range is 5.79 and 5.46 years. That range was reduced for pulsating flow with 10 Hz to be 5.46 and 4.83. Fewer years are observed for 6 Hz. The minimum values for EPBT based on energy for 4 Hz. EPBT based on exergy has values between 24.17 and 31.93 years for pulsating and continuous flow.

The lowest values for EPBT are observed for pulsating flow, which means that continuous flow takes more time to return the money. Also, it is concluded that a higher frequency isn’t a good choice from the point of view of EPBT, as it takes a longer time to return money. The EPBT based on exergy is usually more than the energy one, as less value is noted for the exergy of the solar system. Although embodied energy is higher for pulsating cases because of the cost of the pulsating valve and energy consumed in it, less EPBT is shown for it because of the larger amount of energy produced with it. It was found that pulsating flow reduced the EPBT based on energy and exergy analysis to 4.34, 24.17 years, respectively.

**Table 11:** Embodied energy and cost of each component used in the solar system.

Collectors Parts	Total Weight (kg)	Total Cost (USD)	Cost%	Embodied energy (kWh/m^2^)
**Heat Transfer absorber plate**	10.5	130	52	650
**Rubber Components**	1.5	5	2	75
**Tube Clips**	0.5	5	2	16
**Fasteners**	1.5	10	4	48
**Fan coil unit**	15	70	28	655
**Support frame**	6	20	8	70
**glass**	10	10	4	16
**Total**	75	250	100	1530

**Table 12:** The energy matrices values for continuous and pulsating flow for continuous and pulsating flow.

Case	flow rate (L/h)	Embodied energy (kWh/m^2^)	En out (kWh/m^2^) annual	Ex out (kWh/m^2^) annual	EPBT energy	EPBT exergy
**Continuous flow**	360	2351	406	88.35	5.79	26.61
	420	2351	421	75.32	5.58	31.21
	480	2351	430	73.64	5.47	31.93
**Pulsating flow 10 Hz**	360	2354.7	425	91.88	5.54	25.63
	420	2354.7	466	80.20	5.05	29.36
	480	2354.7	488	74.90	4.83	31.44
**Pulsating flow 6 Hz**	360	2353.2	483	95.51	4.87	24.64
	420	2353.2	496	82.62	4.74	28.48
	480	2353.2	525	79.67	4.48	29.54
**Pulsating flow 4 Hz**	360	2352.47	502	97.35	4.69	24.17
	420	2352.47	535	85.22	4.40	27.61
	480	2352.47	542	80.85	4.34	29.10

## Comparison with previous work

**Fig. 5:**
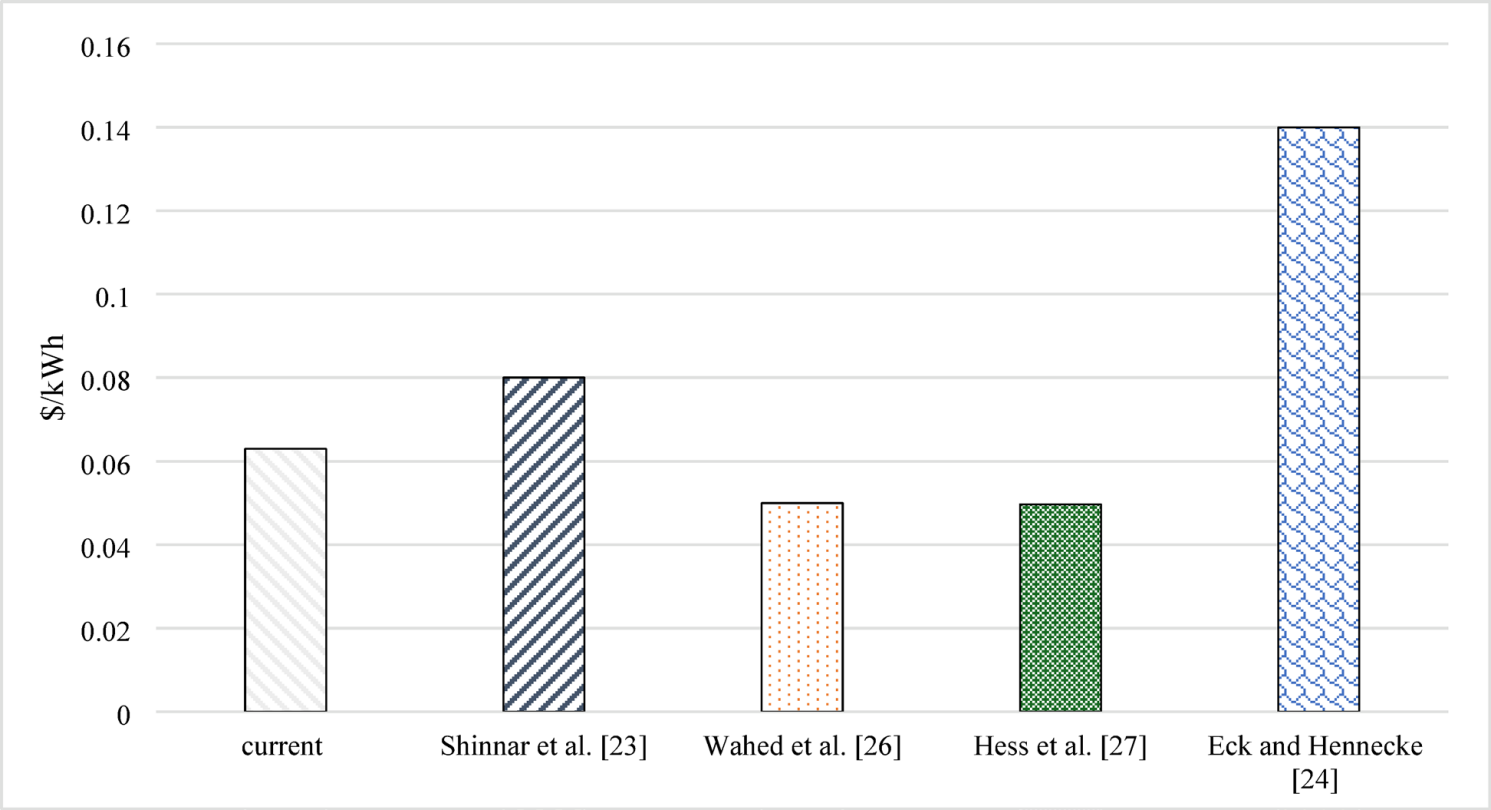
Comparison between the current work and the previous work based on the price of energy.

**Fig. 6:**
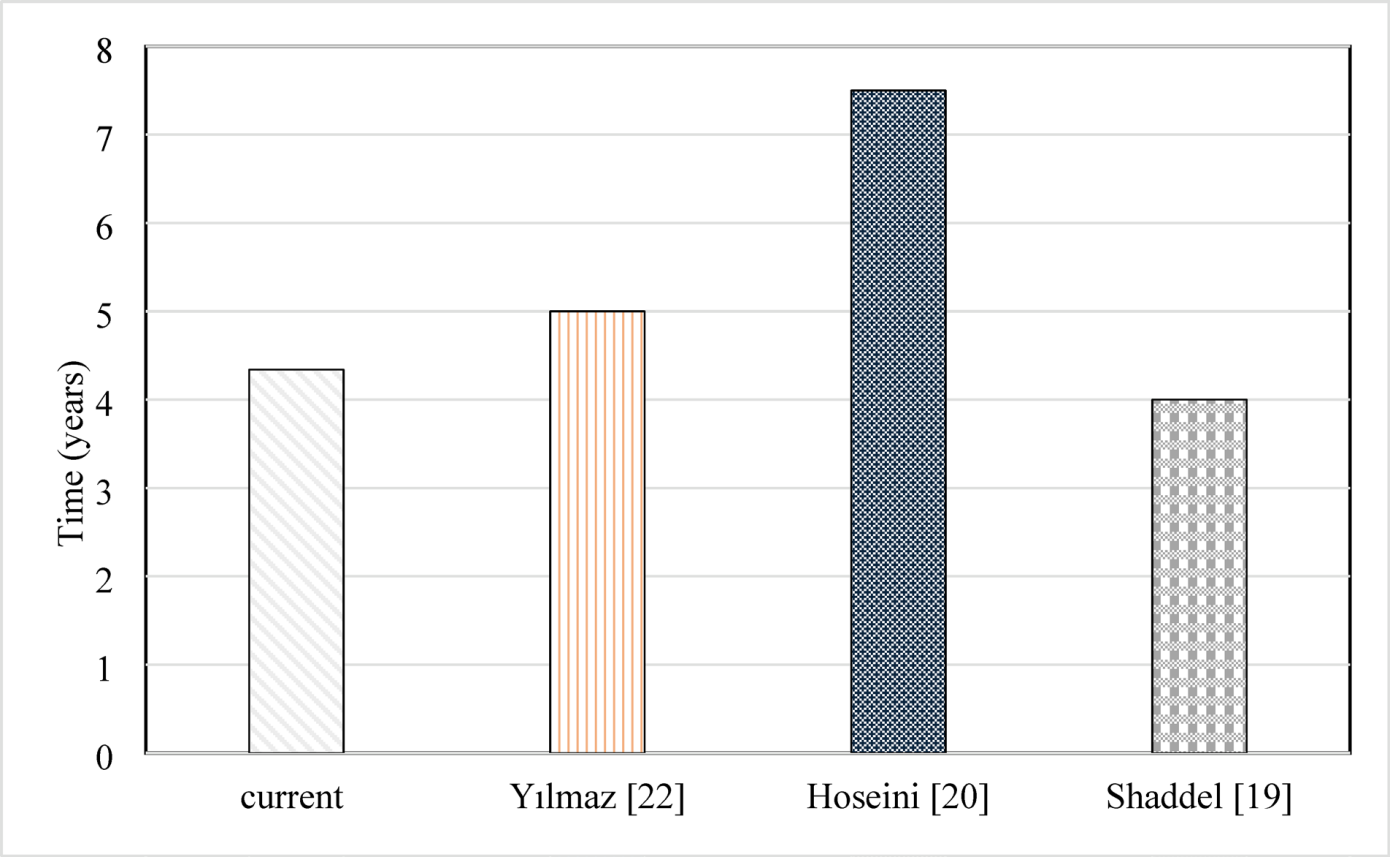
Comparison between the current work and previous work-based energy payback period.

The evidence of the importance of the research appears in its ability to compete compared to previous research. The presented idea will not be of great importance unless it is compared to previous ideas. Accordingly, a detailed comparison of all the important points and results of this research with previous research was conducted. The price is the main issue driving the investment in renewable energy enhancement techniques. Also, the energy payback period is another important parameter to encourage the use of enhancement techniques. The comparison between results from the current work and previous work based on the price of the produced energy is shown in Figure 5. The payback period comparison is shown in Figure 6. The price of energy was $0.0497/kWh for Hess et al.^27^. Shinnar et al.^23^, Wahed et al.^26^,, and Eck and Hennecke^24^ showed that the price of each kW can be 0.08, 0.05, and 0.14 $/kWh. The energy payback periods of Hoseini^20^, Yilmaz^22^, and Shaddel^19^ were 7.5, 5, and 4 years, respectively. According to Figs. 5 and 6, the current has a competitive price and payback period compared with previous studies.

## Conclusion

Solar energy is a fundamental component of the clean energy principles of global sustainability. Thus, the main goal of researchers is to figure out how to improve the performance of solar collectors. Thus, the goal of this study is to examine the effects of pulsating flow on solar collectors through the use of energy, economic, and environmental studies. A thorough test system was used for the measurements. The fluid’s temperature, pressure, flow rate, exposure to sunlight, and ambient temperature were all measured. Entropy generation and energy efficiency were computed. The cost of the heat generated by the system’s design was established. Estimates of greenhouse gas emissions avoided were made. The calculation of exergoeconomic using the energy concept was determined. The energy payback time based on energy and exergy was evaluated. The energy production factor based on energy was computed. The results of the current work can be summarized as.

- Exergy destruction decreases with the reduction of the frequency of pulsating flow.
- Entropy generation increased with the increase of pulsating frequency.
- Compared to continuous flow, pulsating flow has a higher energy efficiency.
- The lower frequency showed higher exergy efficiency.
- Exergy efficiency reached 9.33% for 4 Hz at flow rate of 360 L/hr.
- The greater the frequency, the cheaper the energy cost.
- A notable rise in energy output when low-frequency pulsing flow is present.
- The energy price was lowered by 23.2% in the circumstances under study.
- The enhancement ratio of exergoeconomic (energy) was 31%.
- The net CO₂ emission mitigation reached 13.4 tons/year for the pulsating flow of 4 Hz.
- The EPBT based on energy lowered to 4.34 years in the case of using pulsating flow.
- The EPBT based on exergy dropped to 24.17 years.

### Future work

The use of artificial neural networks (ANNs) is recommended as a future direction for this work. Researchers aim to achieve more accurate and realistic predictions by training and applying suitable algorithms.

## Data Availability

All data generated or analysed during this study are included in this published article.

## List of symbols

A_c_: Solar collector area (m²)
*C*_*p*_: Specific heat (J/kg, K)
$$\dot{E}$$: exergy
F_R_: Heat removal factor
G_T_: Normal solar radiation (W/m²)
h: Enthalpy (J/kg)
C_P_: Heat capacity (J/kg. K)
$\:\dot{\text{m}\:}$: mass flow rate (kg/sec)
*P*: Pressure (pa)
Q_u_: Useful rate of heat energy (W)
$$\:{\stackrel{\prime }{Q}}_{s}$$: Sun radiation (W)
T_a_: Ambient temperature (K)
T_i_: Collector inlet temperature (K)
T_o_: Collector outlet temperature (K)
T_s_: Apparent sun temperature (K)
T_g_: Glass temperature (K)
s: Entropy
U_L_: Overall coefficient of heat loss (W/m^2^- K)
U_max_: maximum values of velocities for the pulsing condition (m/s)
$\:{\text{V}}^{\cdot\:}$: Volume flow rate (m³/hr)

## Greek symbols

$\:{\upalpha\:}$: The absorbance of the solar collector
$$\:{{\upeta\:}}_{e}$$: Exergy efficiency
$\:\rho\:$: Density (kg/m³)
$\:{\uptau\:}$: The transmittance of the collector glass
